# Factor Analysis of Health Care Access With Ovarian Cancer Surgery and Gynecologic Oncologist Consultation

**DOI:** 10.1001/jamanetworkopen.2022.54595

**Published:** 2023-02-01

**Authors:** Anjali Gupta, Quan Chen, Lauren E. Wilson, Bin Huang, Maria Pisu, Margaret Liang, Rebecca A. Previs, Haley A. Moss, Kevin C. Ward, Maria J. Schymura, Andrew Berchuck, Tomi F. Akinyemiju

**Affiliations:** 1 Department of Population Health Sciences, Duke University School of Medicine, Durham, North Carolina; 2 Department of Biostatistics and Kentucky Cancer Registry, University of Kentucky, Lexington; 3 O’Neal Comprehensive Cancer Center, Division of Preventive Medicine, University of Alabama at Birmingham, Birmingham; 4 Division of Gynecologic Oncology, Department of Obstetrics & Gynecology, University of Alabama at Birmingham, Birmingham; 5 Duke Cancer Institute, Division of Gynecologic Oncology, Duke University School of Medicine, Durham, North Carolina; 6 Labcorp Oncology, Durham, North Carolina; 7 Georgia Cancer Registry, Emory University, Atlanta; 8 New York State Cancer Registry, New York State Department of Health, Albany; 9 Duke Cancer Institute, Duke University School of Medicine, Durham, North Carolina

## Abstract

**Importance:**

Poor health care access (HCA) is associated with racial and ethnic disparities in ovarian cancer (OC) survival.

**Objective:**

To generate composite scores representing health care affordability, availability, and accessibility via factor analysis and to evaluate the association between each score and key indicators of guideline-adherent care.

**Design, Setting, and Participants:**

This retrospective cohort study used data from patients with OC diagnosed between 2008 and 2015 in the Surveillance, Epidemiology, and End Results (SEER) Medicare database. The SEER Medicare database uses cancer registry data and linked Medicare claims from 12 US states. Included patients were Hispanic, non-Hispanic Black, and non-Hispanic White individuals aged 65 years or older diagnosed from 2008 to 2015 with first or second primary OC of any histologic type (*International Classification of Diseases for Oncology, 3rd Edition* [*ICD-O-3*] code C569). Data were analyzed from June 2020 to June 2022.

**Exposures:**

The SEER-Medicare data set was linked with publicly available data sets to obtain 35 variables representing health care affordability, availability, and accessibility. A composite score was created for each dimension using confirmatory factor analysis followed by a promax (oblique) rotation on multiple component variables.

**Main Outcomes and Measures:**

The main outcomes were consultation with a gynecologic oncologist for OC and receipt of OC-related surgery in the 2 months prior to or 6 months after diagnosis.

**Results:**

The cohort included 8987 patients, with a mean (SD) age of 76.8 (7.3) years and 612 Black patients (6.8%), 553 Hispanic patients (6.2%), and 7822 White patients (87.0%). Black patients (adjusted odds ratio [aOR], 0.75; 95% CI, 0.62-0.91) and Hispanic patients (aOR, 0.81; 95% CI, 0.67-0.99) were less likely to consult a gynecologic oncologist compared with White patients, and Black patients were less likely to receive surgery after adjusting for demographic and clinical characteristics (aOR, 0.76; 95% CI, 0.62-0.94). HCA availability and affordability were each associated with gynecologic oncologist consultation (availability: aOR, 1.16; 95% CI, 1.09-1.24; affordability: aOR, 1.13; 95% CI, 1.07-1.20), while affordability was associated with receipt of OC surgery (aOR, 1.08; 95% CI, 1.01-1.15). In models mutually adjusted for availability, affordability, and accessibility, Black patients remained less likely to consult a gynecologic oncologist (aOR, 0.80; 95% CI, 0.66-0.97) and receive surgery (aOR, 0.80; 95% CI, 0.65-0.99).

**Conclusions and Relevance:**

In this cohort study of Hispanic, non-Hispanic Black, and non-Hispanic White patients with OC, HCA affordability and availability were significantly associated with receiving surgery and consulting a gynecologic oncologist. However, these dimensions did not fully explain racial and ethnic disparities.

## Key Points

**Question:**

Are health care affordability, availability, and accessibility associated with racial and ethnic disparities in consulting a gynecologic oncologist and receiving surgery for ovarian cancer?

**Findings:**

In this cohort study of 8987 patients, health care availability and affordability scores were associated with gynecologic oncologist consultation, while affordability scores were associated with surgery receipt. However, non-Hispanic Black patients were less likely than non-Hispanic White patients to consult a gynecologic oncologist and have surgery even after adjusting for these health care access factors.

**Meaning:**

These findings suggest that affordability and availability were significantly associated with indicators of guideline-adherent care but did not fully explain disparities.

## Teaser

This cohort study assesses health care affordability, availability, and accessibility along with race and ethnicity in association with receipt of guideline-adherent care for ovarian cancer.

## Introduction

Ovarian cancer (OC) mortality in the United States declined 33% from 1976 to 2015; however, not all racial and ethnic groups have benefited equally.^1^ Although 5-year OC survival in White patients has improved from 35% for those diagnosed from 1975 to 1977 to 48% for those diagnosed from 2011 to 2017, 5-year survival in Black patients has remained constant, at approximately 40%.^1,2^ Lower-quality care among Black patients likely contributes to these disparities.

Multiple studies have documented lower rates of guideline-concordant treatment among Black patients.^3,4,5,6,7^ However, equal survival among racial groups has been documented with equal receipt of treatment,^3,4,6^ suggesting that racial disparities in OC survival may be associated with differences in access to quality health care. Key indicators of guideline-adherent treatment include consultation with a gynecologic oncologist and stage-appropriate surgery performed by a gynecologic oncologist.^4,8,9^ Knowledge of factors that impede access to high-quality care for all racial groups is crucial to mitigating survival disparities for Black patients with OC.

Penchansky and Thomas^10^ proposed a model of health care access (HCA) focused on 5 separate but related dimensions: affordability (ability to pay), availability (type, quality, and volume of services), accessibility (geographic location of services), acceptability (patient experience and quality of patient-clinician interaction), and accommodation (organization of resources in relation to the patient’s needs), outlined in eTable 1 in Supplement 1. Components of these HCA dimensions are associated with receipt of guideline-adherent treatment for OC.^11^ For example, low socioeconomic status (SES) (affordability),^11,12,13^ lower-volume facilities (availability),^11,13^ and nonprivate insurance (affordability)^14^ are associated with lower likelihood of guideline-adherent treatment. However, adjustment for many individual measures representing a single HCA dimension (eg, household poverty, education, and insurance for affordability) reduces statistical power and may not capture potential additive associations of these measures. Collinearity among variables measuring the same dimension further reduces the precision of estimated coefficients. To address these issues, the objectives of this study were to use factor analysis to generate scores representing health care affordability, availability, and accessibility; describe the methods for creation of the HCA scores; and evaluate the associations between each score and 2 key indicators of guideline-adherent care—receipt of surgery and consultation with a gynecologic oncologist—among Hispanic, non-Hispanic Black, and non-Hispanic White patients with OC.

## Methods

This cohort study was approved by the institutional review board of Duke University. Because this was a Surveillance, Epidemiology, and End Results (SEER)–Medicare secondary analysis, there was no need for informed consent, per the Duke University institutional review board and National Institutes of Health policy. This study followed the Strengthening the Reporting of Observational Studies in Epidemiology (STROBE) reporting guideline for cohort studies.

### Study Population

Black, Hispanic, and White patients aged 65 years or older diagnosed from 2008 to 2015 with first or second primary OC of any histologic type (*International Classification of Diseases for Oncology, 3rd Edition* [*ICD-O-3*] code C569) were selected from the SEER-Medicare linked data set, which combines cancer registry data from 12 US states. Cohort selection is detailed in eFigure 1 in Supplement 1. Patients were excluded if they were diagnosed at autopsy or death or if OC was not their first or second primary tumor in the SEER registry. Patients with cancers originating from the fallopian tubes or peritoneum were not included (<11 patients). Patients with at least 12 months of continuous enrollment in Medicare fee-for-service parts A and B prior to diagnosis were included to enable assessment of patient comorbid conditions. All included patients had at least 1 Medicare claim with a diagnosis code for OC (*International Classification of Diseases, Ninth Revision, Clinical Modification *[*ICD-9-CM*] and *International Statistical Classification of Diseases, Tenth Revision, Clinical Modification *[*ICD-10-CM*] diagnosis codes 183.0 or C569) within 2 months of the SEER diagnosis. This effectively limited our cohort to patients aged 66 years or older at OC diagnosis and patients who were eligible for Medicare before age 65 years due to disability or a qualifying medical condition. Patients were required to have continuous fee-for-service Medicare enrollment in the 12 months following their diagnosis date or until death for assessment of treatment receipt. There were 9726 patients initially; patients with any missing HCA dimension variable values were excluded, resulting in 8987 patients eligible for analysis.

### Demographic and Clinical Characteristics

We sourced patient demographic and clinical characteristics from the SEER Program, including race and ethnicity, age at diagnosis, cancer stage at diagnosis, histology (type I epithelial, type II epithelial, or other),^15^ marital status, and geographic region of residence. Race and ethnicity in SEER are drawn from medical records and administrative data; a validation study comparing SEER data to self-reported race and ethnicity found excellent agreement for race variables (κ > 0.90) and good agreement for Hispanic ethnicity (κ = 0.63).^16^ We used validated coding algorithms to calculate the Charlson Comorbidity Index in the 12 months prior to the patient’s OC diagnosis date using diagnosis codes (eAppendix 1 in Supplement 1) from inpatient, outpatient, and carrier Medicare claims.^17,18^

### Metrics of Health Care Access Affordability, Accessibility, and Availability

To assess measures of each patient’s affordability, accessibility, and availability, SEER-Medicare data were linked with publicly available data sets from the US Census Bureau, the Small Area Health Insurance Estimates Program, the Dartmouth Atlas of Healthcare, and the Area Healthcare Resource Files provided by the Health Resources and Services Administration .^19^ In total, 35 variables were selected across the 3 dimensions of affordability, availability, and accessibility. Full detail on these measures is available in the eMethods in Supplement 1. Area-level statistics (census tract, county, and hospital referral region) were based on the patient’s residence at diagnosis, and, when possible, specific to the year of diagnosis. Descriptive analyses were performed to calculate the mean, median, SD, and IQR as appropriate for continuous variables, and frequency and percentage for categorical variables.

### Outcome Measures

Outcome measures of interest were access to a gynecologic oncologist and receipt of ovarian cancer surgery. All patients who consulted a gynecologic oncologist for their OC at least once in the 2 months prior to or in the 6 months after diagnosis were identified from inpatient, outpatient, and carrier claims and their Health Care Finance Administration (HCFA) specialty codes. If the patient had a claim with a physician with a specialty code indicating “gynecologist/oncologist” (HCFA code 98), they were considered to have had at least 1 encounter. HCFA specialty codes capture approximately 83% to 90% of oncologists.^20^ Receipt of OC-related surgery in the 2 months prior to or the 6 months after diagnosis was defined consistent with previous studies.^21^ If a patient had a claim with any of the OC-related surgical procedure codes (eAppendix 2 in Supplement 1) in that time period, they were considered to have received surgery.

### Statistical Analysis

We used the Penchansky and Thomas framework^10^ to guide our selection of variables representing the hypothesized latent constructs of HCA (affordability, availability, and accessibility) to include in our analysis using a 2-stage confirmatory factor analysis (CFA) approach. First, factor analysis was conducted for each a priori grouping of HCA dimension measures (affordability, availability, and accessibility), then variables with loadings higher than 0.5 in the 3 preliminary models were carried forward into 1 final combined model. Two variables measuring number of specialists available (gynecologic oncologists and obstetrician gynecologists) had high correlation efficiency, thus the obstetrician gynecologist variable was excluded. A total of 18 HCA dimension measures were loaded into the second-stage factor analysis. There was a clear separation for each of the 3 hypothesized factors on the factor analysis scree plot (eFigure 2 in Supplement 1), and each factor captured most of the measures for a hypothesized HCA domain. We next conducted reliability tests and assessed model fit for these selected factors (eTable 2 in Supplement 1). Based on the reliability tests, we adjusted our final factor model by excluding the number of hospitals per 1000 county population variable, which resulted in improved reliability metrics for the accessibility domain. The final factor model comprised a total of 13 variables loading onto the 3 factors, with close to 89% of the sample variance explained. To assess whether alternate factor structures would provide better explanation of the data variability, we conducted parallel exploratory factor analyses unsupervised and with different numbers of latent factors defined to agnostically determine factor structure. A 3 factor model was produced, but this model had low reliability scores for 2 of the produced factors when assessed using the composite reliability (eTable 3 in Supplement 1). Of all models assessed, the 2-staged confirmatory factor model performed the best on standard reliability testing and fit assessment. Methods and results from the full parallel analyses to inform the maximum number of factors to rotate are included in the eMethods and eTable 4 in Supplement 1. We also performed an invariance assessment across racial and ethnic groups; the model fit measures across all groups and combined were similarly good (eTable 5 in Supplement 1).

Estimated factor scores for each HCA domain were created using PROC FACTOR and PROC SCORE to generate a linear composite of optimally weighted variables under analysis. Standardized factor-weighted sum scores were compared for each factor across patient race and ethnicity. Scores ranged from approximately −3 to 4, with lower values representing lower HCA (eg, low affordability). Multivariable logistic regression was performed to analyze associations of factor scores (modeled as 1-unit change) with 2 outcome variables: whether the patient consulted a gynecologic oncologist and whether they received any surgery, while adjusting for patient race and ethnicity and other clinical and demographic variables, including age, stage, grade, and histology. Interactions between the factor scores and race and ethnicity were evaluated. Patients with missing values were excluded from analysis. All statistical tests were 2-sided with *P* < .05 denotating significance. All analyses were conducted using SAS version 9.4 (SAS Institute) and Mplus version 8.0 (Muthén and Muthén). Data were analyzed from June 2020 to June 2022.

## Results

Among 8987 eligible patients, 553 (6.2%) were Hispanic, 612 (6.8%) were non-Hispanic Black, and 7822 (87.0%) were non-Hispanic White. Overall, the mean (SD) age at diagnosis was 76.8 (7.3) years. White patients had the highest rates of Type II epithelial OC (6423 patients [82.1%]); Black patients had higher median (IQR) comorbidity scores (3.0 [2.0-5.0]) than White patients (2.0 [1.0-4.0]) and were more likely to be initially diagnosed with stage IV cancer (247 patients [40.4%] vs 2540 patients [32.5%]) (Table 1). Hispanic patients were more similar clinically to White patients, with the exception of higher median (IQR) comorbidity scores (3.0 [1.0-5.0]). Most HCA dimension variables differed across race and ethnicity (Table 2). For example, Black and Hispanic patients were more likely to be dual-enrolled in Medicaid and Medicare than White patients (255 Black patients [41.7%] and 273 Hispanic patients [49.4%] vs 870 White patients [11.2%]) and more likely to live in metropolitan or metropolitan-adjacent areas (600 Black patients [98.0%] and 535 Hispanic patients [96.8%] vs 7280 White patients [93.1%]).

**Table 1.** Patient Demographic and Clinical Characteristics Stratified by Race and Ethnicity

Variable	Patients, No. (%) (N = 8987)		
	Hispanic (n = 553)	Non-Hispanic	
		Black (n = 612)	White (n = 7822)
Age at OC diagnosis, mean (SD), y	75.7 (6.9)	76.0 (6.9)	77.0 (7.4)
Married	185 (33.5)	125 (20.4)	3318 (42.4)
Geographic region			
Midwest	18 (3.3)	91 (14.9)	1017 (13.0)
Other or missing	<11	75 (12.3)	681 (8.7)
Northeast	84 (15.2)	141 (23.0)	1737 (22.2)
South	>15	199 (32.5)	1165 (14.9)
West	428 (77.4)	106 (17.3)	3222 (41.2)
Comorbidity score, median (IQR)	3.0 (1.0-5.0)	3.0 (2.0-5.0)	2.0 (1.0-4.0)
Comorbidities			
Myocardial infarction	28 (5.1)	50 (8.2)	413 (5.3)
Hypertension	437 (79.0)	550 (89.9)	5900 (75.4)
Peripheral vascular disease	114 (20.6)	162 (26.5)	1480 (18.9)
Congestive heart failure	110 (20.0)	154 (25.2)	1218 (15.6)
Dementia	32 (5.8)	41 (6.7)	269 (3.4)
Cardiovascular disease	84 (15.2)	119 (19.4)	1234 (15.8)
COPD	140 (25.3)	159 (26.0)	1970 (25.2)
Rheumatologic disease	48 (8.7)	43 (7.0)	472 (6.03)
Peptic ulcer disease	21 (3.8)	23 (3.8)	225 (2.9)
Mild liver disease	116 (21.0)	101 (16.5)	1157 (14.8)
Kidney disease	81 (14.7)	121 (19.8)	889 (11.4)
Any diabetes	244 (44.1)	260 (42.5)	1994 (25.5)
Diabetes with complications	97 (17.5)	97 (15.9)	496 (6.3)
Hemiplegia or paraplegia	<11	<15	87 (1.1)
OC care received			
Any gynecologic oncologist consultation	259 (46.8)	276 (45.1)	4288 (54.8)
Received any cancer-directed surgery	329 (59.5)	308 (50.3)	4824 (61.7)
Cancer sequence			
First primary	499 (90.2)	562 (91.8)	6858 (87.7)
Second primary	54 (9.8)	50 (8.2)	964 (12.3)
Tumor stage at diagnosis			
I	60 (10.9)	64 (10.5)	904 (11.6)
II	32 (5.8)	36 (5.9)	521 (6.7)
III	190 (34.4)	185 (30.2)	2887 (36.9)
IV	190 (34.4)	247 (40.4)	2540 (32.5)
Unknown	81 (14.7)	80 (13.1)	970 (12.4)
Histology			
Type I epithelial	77 (13.9)	63 (10.3)	869 (11.1)
Type II epithelial	434 (78.5)	479 (78.3)	6423 (82.1)
Other	42 (7.6)	70 (11.4)	530 (6.8)
Year of diagnosis			
2008	73 (13.2)	88 (14.4)	1156 (14.8)
2009	65 (11.8)	76 (12.4)	1075 (13.7)
2010	75 (13.6)	78 (12.8)	1019 (13.0)
2011	63 (11.4)	84 (13.7)	906 (11.6)
2012	69 (12.5)	91 (14.9)	905 (11.6)
2013	69 (12.5)	79 (12.9)	928 (11.9)
2014	76 (13.7)	60 (9.8)	905 (11.6)
2015	63 (11.4)	56 (9.2)	928 (11.9)

Abbreviations: COPD, chronic obstructive pulmonary disease; OC, ovarian cancer.

**Table 2.** Baseline Patient Measures of Health Care Affordability, Accessibility, and Availability at Time of Ovarian Cancer Diagnosis by Race and Ethnicity

Measure	Patients (N = 8987)		
	Hispanic (n = 553)	Non-Hispanic	
		Black (n = 612)	White (n = 7822)
**Affordability **			
Categorical variables, No. (%)			
Patient is dual-enrolled in Medicaid and Medicare	273 (49.4)	255 (41.7)	870 (11.2)
Patient’s primary hospital eligibility for disproportionate share payments	481 (87.0)	518 (84.6)	5853 (74.8)
Continuous variables, mean (SD)			
Census tract–level variable at diagnosis			
Black residents, %	6.1 (9.9)	50.6 (33.2)	7.6 (13.3)
Persons aged ≥25 y with ≥4 y of college, %	24.4 (17.6)	20.4 (14.8)	33.0 (18.9)
Median household income, $	57 021.6 (26 982.3)	45 296.9 (23 609)	67 757.9 (31 658.8)
Persons aged ≥25 y with <high school education	22.7 (16.4)	19.5 (10.7)	11.6 (9.1)
Per capita income for Census tract, $	26 577.1 (14 668.5)	22 026.4 (9964.6)	34 327.4 (17 108.3)
Persons aged ≥25 with some college education	28.4 (8.7)	28.6 (7.8)	29.3 (7.9)
Households below poverty line, %	17.5 (11.7)	23.0 (13.3)	11.8 (9.2)
County-level residents without health insurance, %	16.2 (5.1)	15.9 (4.6)	14.0 (5.0)
**Accessibility**			
Categorical variables, No. (%)			
Patient lives in metropolitan area	>500	>540	6535 (83.6)
Patient lives in a metropolitan or metropolitan-adjacent area	535 (96.8)	600 (98.0)	7280 (93.1)
Patient lives in rural area	<11	<11	174 (2.2)
Patient’s main hospital is rural primary hospital	<11	13 (2.1)	364 (4.7)
Continuous variables, mean (SD)			
Straight-line geographic distance from patient residential zip code to patient’s main hospital zip code, mi	20.1 (125.4)	21.5 (150.2)	21.2 (119.0)
County-level hospitals per 1000 residents in patient’s county in year of diagnosis, No.	1.5 (1.6)	2.2 (2.1)	2.0 (2.1)
**Availability**			
Categorical variables, No. (%)			
Patient’s main hospital teaching status	295 (53.4)	400 (65.4)	3941 (50.4)
Patient’s main hospital NCI cancer center designation			
Clinical	25 (4.5)	<11	178 (2.3)
Comprehensive	33 (6.0)	<50	507 (6.5)
Patient’s main hospital is member of NCI gynecologic oncology group	108 (19.5)	139 (22.7)	1629 (20.8)
Specialty of patient’s PCP			
General surgery	<11	<11	97 (1.2)
Gynecologic oncology	112 (20.3)	146 (23.9)	1803 (23.1)
Hematology, oncology, or medical oncology	258 (46.7)	220 (36.0)	3629 (46.4)
Internal medicine	73 (13.2)	86 (14.1)	793 (10.1)
No primary	20 (3.6)	29 (4.7)	184 (2.4)
Obstetrician gynecologist	30 (5.4)	49 (8.0)	446 (5.7)
Other	20 (3.6)	42 (6.9)	324 (4.1)
Pathology or other oncology	<20	<11	99 (1.3)
Primary or general	23 (4.2)	24 (3.9)	430 (5.5)
Surgical oncology	0	0	17 (0.2)
Continuous variables, mean (SD)			
Beds in patient’s main hospital, No.	332.6 (202.2)	395.2 (253.3)	429.0 (4467.0)
HRR-level factors			
Discharges for ambulatory sensitive conditions, No. per 1000 population	51.4 (13.7)	65.7 (16.5)	57.5 (18.4)
Hematologists or oncologists, No. per 100 000 residents	3.1 (0.8)	3.3 (0.9)	3.3 (0.9)
Medicare beneficiaries who died in year of diagnosis, %	4.0 (0.5)	4.6 (0.6)	4.4 (0.6)
Hospital-based physicians, No. per 100 000 residents^a^	25.6 (2.4)	24.4 (2.6)	25.6 (2.8)
Obstetrician gynecologists, No. per 100 000 women	56.5 (14.8)	60.3 (12.0)	60.2 (14.9)
Medicare beneficiaries seeing a PCP that year, %	73.9 (4.6)	77.2 (5.0)	77.1 (4.8)
PCPs per 100 000 residents, No.	73.9 (12.1)	72.1 (9.6)	74.7 (11.2)
Hospital discharge 30 d return to ED rate, %	19.3 (1.5)	19.7 (1.3)	19.6 (1.4)
30 d hospital readmission rates, %	15.4 (1.2)	16.1 (1.0)	15.5 (1.2)
Physicians, No. per 100 000 residents	206.4 (32.0)	206.8 (28.5)	210.6 (30.5)
Surgeons, No. per 100 000 residents	40.1 (7.0)	41.6 (5.4)	42.1 (6.4)
County-level factors, No. per 1000 residents			
Gynecologic oncologists in year of diagnosis	11.9 (5.9)	15.4 (8.4)	12.1 (7.4)
Obstetric gynecologists seeing patients	11.6 (5.7)	14.9 (8.2)	11.8 (7.2)
PCPs	72.6 (24.0)	77.2 (26.4)	76.0 (28.0)

Abbreviations: ED, emergency department; HRR, hospital referral region; NCI, National Cancer Institute; PCP, primary care physician.

^a^ Data from 2011.

Results of the combined factor analysis are presented in Table 3 and eTable 6 in Supplement 1. Based on HCA measure loadings, factor 1 represents availability, with 5 measures selected; factor 2 represents affordability, with 5 measures selected; and factor 3 represents accessibility, with 4 measures selected. White patients had higher median (IQR) scores for availability (−0.109 [−0.657 to 0.737]) and affordability (−0.001 [−0.551 to 0.661]) than Black patients (availability: −0.288 [−0.688 to 0.577]; affordability: −0.842 [−1.267 to −0.285]) and Hispanic patients (availability: −0.141 [−0.278 to 0.477] affordability: −0.579 [−1.144 to 0.119]) (Table 4). For accessibility, White patients had lower median (IQR) scores (0.169 [0.058 to 0.278]) than Black patients (0.194 [0.117 to 0.287]) and Hispanic patients (0.263 [0.136 to 0.362]), indicating lower accessibility.

**Table 3.** Factor Loadings From the 2-Stage 3-Factor CFA Solution

Factor^a^	Standardized factor loadings
**Availability: HRR-level variables**	
Hematologists or oncologists per 100 000 residents^b^	0.76
Hospital-based physicians per 100 000 residents^b^	0.63
HRR: Primary care physicians per 100 000 residents^b^	0.88
HRR: Total physicians per 100 000 residents^b^	1.00
HRR: Surgeons per 100 000 residents^b^	0.75
**Affordability: census tract–level variables at diagnosis**	
Census tract at diagnosis: % residents 25+ with at least 4 y of college	0.88
Census tract at diagnosis: median household income	0.85
Census tract at diagnosis: % residents 25+ with <12 y education	−0.83
Census tract at diagnosis: mean per capita income	0.89
Census tract at diagnosis: % households below poverty line	−0.74
**Accessibility variables**	
Patient residence in a metropolitan or metropolitan-adjacent area	0.63
Patient lives in metropolitan area	0.78
Patient’s main hospital is designated rural primary hospital	−0.52
County-level hospitals, No. per 1000 residents in year of diagnosis	−0.67

Abbreviations: CFA, confirmatory factor analysis; HRR, hospital referral region.

^a^ Model fit: comparative fit index: 0.90; Tucker-Lewis Index: 0.88; standardized root-mean-square residual: 0.07.

^b^ Data from 2011.

**Table 4.** Factor Scores by Patient Race and Ethnicity

Factor	Factor scores, median (IQR)		
	Hispanic	Non-Hispanic	
		Black	White
Availability score	−0.141 (−0.278 to 0.477)	−0.288 (−0.688 to 0.577)	−0.109 (−0.657 to 0.737)
Affordability score	−0.579 (−1.144 to 0.119)	−0.842 (−1.267 to −0.285)	−0.001 (−0.551 to 0.661)
Accessibility score	0.263 (0.136 to 0.362)	0.194 (0.117 to 0.287)	0.169 (0.058 to 0.278)

Racial and ethnic differences were further observed in consultation with a gynecologic oncologist and receipt of surgery; White patients had the highest prevalence of both indicators of guideline-adherent care compared with Black and Hispanic patients: 4288 White patients (54.8%) received a consultation with a gynecologic oncologist, compared with 276 Black patients (45.1%) and 259 Hispanic patients (46.8%), and 4824 White patients (61.7%) underwent cancer-directed surgery, compared with 308 Black patients (50.3%) and 329 Hispanic patients (59.5%) (Table 1). Black patients were less likely to consult a gynecologic oncologist than White patients (adjusted odds ratio [aOR], 0.75; 95% CI, 0.62 to 0.91) after adjusting for patient demographic and clinical characteristics (Table 5). Availability and affordability scores were each statistically significant factors associated with receipt of gynecologic oncologist consultation (Table 5), with higher scores associated with a higher likelihood of consultation (availability: aOR, 1.16; 95% CI, 1.09 to 1.24; affordability: aOR, 1.13; 95% CI, 1.07 to 1.20). In addition, affordability score was associated with the patient’s receipt of cancer-directed surgery (aOR 1.08; 95% CI, 1.01 to 1.15) (Table 5). No associations were found for availability or accessibility scores and receipt of cancer-directed surgery. In models mutually adjusted for availability, affordability, and accessibility, Black patients remained less likely to receive a gynecologic oncologist consultation (aOR, 0.80; 95% CI, 0.66 to 0.97) or surgery (aOR, 0.80; 95% CI, 0.65 to 0.99) than White patients. In interaction analyses, the only interaction that was statistically significant was the interaction between Hispanic ethnicity and accessibility for the association with seeing a gynecologic oncologist (β = −0.37; SE, 0.18; *P* = .04).

**Table 5.** Logistic Regression Analysis of Associations With Patient Visiting a Gynecologic Oncologist at Least Once in the 2 Months Prior to or 6 Months Following Diagnosis of Ovarian Cancer and With Patient Received Any Cancer-Directed Surgery

Variable	aOR (95% CI)				
	Demographic/clinical^a^	Demographic/clinical + availability score^a^	Demographic/clinical + affordability score^a^	Demographic/clinical + accessibility score^a^	Demographic/clinical + all 3 scores^a^
**Patient visited gynecologic oncologist**					
Race and ethnicity					
Hispanic	0.81 (0.67-0.99)	0.84 (0.69-1.02)	0.88 (0.72-1.07)	0.81 (0.67-0.98)	0.89 (0.73-1.09)
Non-Hispanic Black	0.75 (0.62-0.91)	0.75 (0.62-0.90)	0.81 (0.67-0.98)	0.74 (0.62-0.90)	0.80 (0.66-0.97)
Non-Hispanic White	1 [Reference]	1 [Reference]	1 [Reference]	1 [Reference]	1 [Reference]
Availability score	NA	1.16 (1.09-1.24)	NA	NA	1.14 (1.07-1.21)
Affordability score	NA	NA	1.13 (1.07-1.20)	NA	1.11 (1.04-1.17)
Accessibility score	NA	NA	NA	1.05 (0.96-1.16)	1.00 (0.92-1.11)
**Patient received any cancer-directed surgery**					
Race and ethnicity					
Hispanic	0.91 (0.73-1.13)	0.91 (0.73-1.13)	0.95 (0.76-1.19)	0.90 (0.72-1.13)	0.96 (0.76-1.20)
Non-Hispanic Black	0.76 (0.62-0.94)	0.76 (0.62-0.94)	0.80 (0.65-0.98)	0.76 (0.61-0.93)	0.80 (0.65-0.99)
Non-Hispanic White	1 [Reference]	1 [Reference]	1 [Reference]	1 [Reference]	1 [Reference]
Availability score	NA	1.01 (0.94-1.09)	NA	NA	0.99 (0.92-1.07)
Affordability score	NA	NA	1.08 (1.01-1.15)	NA	1.09 (1.02-1.16)
Accessibility score	NA	NA	NA	1.01 (0.91-1.12)	0.98 (0.88-1.09)

Abbreviations: aOR, adjusted odds ratio; NA, not applicable.

^a^ All models adjusted for age at diagnosis, marital status, geographic region, comorbidity score, cancer sequence, tumor stage, histology, and year of diagnosis.

## Discussion

This cohort study used CFA to consolidate measures related to 3 hypothesized dimensions of HCA (affordability, availability, and accessibility) into composite scores representing each dimension. We found that Black and Hispanic patients had lower availability and affordability scores than White patients (indicating poorer availability and affordability), but higher accessibility scores. Additionally, Black patients were less likely to receive surgery or consult a gynecologic oncologist than White patients. Higher affordability score was associated with receipt of surgery, and affordability and availability scores were both associated with gynecologic oncologist consultation.

Our results indicate that better access to health care was associated with guideline-adherent care; these findings are consistent with past studies that have evaluated individual measures of affordability, availability, and accessibility.^22^ The affordability score used in this analysis was primarily comprised of Census tract–level income and education measures. Several studies have shown that lower SES is associated with diminished likelihood of receiving guideline-adherent care for OC.^11,12,13^ Lower SES has also been specifically associated with reduced likelihood of receiving OC surgery.^23,24^ Although research looking at factors associated with gynecologic oncologist consultation is limited, our observed association with affordability is reasonable, as specialist care is known to be cost-prohibitive for some patients, particularly for those who do not have robust insurance coverage.^25,26^ These individuals may delay or avoid seeking care due to cost, leading to worsened conditions and poorer survival.

Furthermore, the availability score, comprised of measures related to the number of physicians, oncologists, and surgeons available in the patient’s referral region, was associated with gynecologic oncologist consultation. Past studies have highlighted hospital type and physician volume as key factors associated with guideline-adherent care. Studies have found that treatment at an National Cancer Institute Comprehensive Cancer Center^27^ or an American College of Surgeons–approved cancer program^28^ is positively associated with receipt of guideline-adherent care. Conversely, treatment at low-volume facilities is negatively associated with guideline-adherent care.^12,13,28^ Our result indicating that greater availability score was associated with higher likelihood of consultation with a gynecologic oncologist is consistent with specialist care being more available in regions with higher physician volume. However, we found no statistically significant associations between accessibility score and surgery receipt or consultation with a gynecologic oncologist. The higher accessibility score observed among Black and Hispanic patients compared with White patients is likely due to the concentration of Black and Hispanic patients in metropolitan areas, where health care resources, including tertiary and academic medical centers, are more likely to be located and, due to population density, be physically closer to patients.^29^ However, the greater local availability of hospitals does not always imply utilization of those resources, especially by patients with low SES and those in minority racial and ethnic groups, such as Black and Hispanic patients,^30^ highlighting the need to evaluate the separate but interrelated dimensions of affordability and accessibility. Where patients receive cancer care is driven by a range of multilevel considerations beyond proximity to health care resources, from cost of care and personal preferences to historical patterns of interactions between academic medical centers and local communities.^22^

We observed that racial disparities in receipt of surgery and consultation with a gynecologic oncologist persisted even after adjusting for a patient’s accessibility, affordability, and availability scores. This is consistent with previous research suggesting that simply addressing disparities in specific SES and access measures, such as income level and insurance coverage, is not sufficient to eliminate racial disparities in OC outcomes.^31,32,33^ Two other HCA dimensions within the Penchansky and Thomas framework^10^ not evaluated here include accommodation (organization of resources in relation to patient’s needs) and acceptability (patient experience and quality of patient-clinician interactions). These measures rely on patient-reported data and are not available in SEER-Medicare data; however, they likely significantly shape utilization of care. Structural racism, implicit bias, patient-clinician communication, and cultural competence are likely major drivers of patient decisions regarding facility choice, adherence to clinician recommendations, and quality of care.^34,35,36,37,38^ Further studies with primary data examining the independent and joint associations of all 5 HCA dimensions with indicators of guideline-adherent care across racial and ethnic groups will be critical to shed more light on these associations. Future studies of disparities could use a similar factor analysis approach leveraging publicly available data to assess HCA dimensions in their own populations.

### Limitations

Our analysis has several limitations. First, as with many analyses using administrative claims data, several HCA dimension measures used were area-level and were not collected yearly; we used the available measures closest to the patient’s date of diagnosis and only used measures for which data were available within 5 years of patients’ diagnoses. These measures may not precisely represent individual patient situations, but they are important in capturing the context in which patients make decisions. Second, we acknowledge that we may have underestimated the number of patients seeing oncologists, as physician specialty codes in Medicare only capture approximately 83% to 90% of oncologists.^20^ Third, this analysis cannot capture the full clinical picture used to drive treatment decisions; some patients with advanced disease may require chemotherapy prior to surgery, which may result in subsequent surgery receipt appropriately occurring more than 6 months after diagnosis, which would not be captured in our analysis, and some patients with metastatic disease may not be recommended for surgery. Likewise, it has been documented that Black patients are more likely to receive neoadjuvant chemotherapy than White patients.^39^ However, adjusting for tumor stage and histology should account for many of these differences. Fourth, our analysis does not capture more personal considerations, such as patient preferences and physician-specific factors, that may have been used to determine care and may not be generalizable to patients in managed care programs.

## Conclusions

In this cohort study of Hispanic, non-Hispanic Black, and non-Hispanic White patients with OC in the SEER-Medicare data set, we observed associations of affordability and availability with surgery receipt and gynecologic oncologist consultation. Multilevel strategies to increase access to care will be crucial to improving outcomes for all patients. These include efforts to enhance individual access, such as more generous insurance policies, and strategies at the hospital and clinician level, such as financial navigation services and partnerships between academic centers and community clinics to increase specialty care in lower-income communities. However, racial disparities persisted in receipt of surgery and consultation with a gynecologic oncologist even after accounting for the HCA scores. Therefore, further research on additional HCA factors also is needed to develop context-specific interventions to improve OC survival among Black patients.

## Notes

Supplement 1.
eMethods.
**eTable 1.** Penchansky Model of Health Care Access Dimensions and Example Measures **eTable 2.** Standardized Factor Loadings and Reliability Test From the 2-Stage 3-Factor Solution for Original and Adjusted Factors **eTable 3.** Factor Loadings and Reliability Test From the Direct 3 Factor Solution (Single-Stage Approach) **eTable 4.** Fit Statistics for All Tested Factor Models **eTable 5.** Invariance Assessment for Model Fit Measures Across Race and Ethnicity Groups for the Final Selected Model **eTable 6.** Factor Loadings With 95% CIs Using the Maximum Likelihood Method **eFigure 1.** Participant Flowchart for Hispanic, Non-Hispanic Black, and Non-Hispanic White Patients With Ovarian Cancer, SEER-Medicare Data Set, 2008-2015 **eFigure 2.** Confirmatory Factor Analysis (CFA) Scree Plots: 2-Stage CFA Approach **eAppendix 1.** Diagnosis Codes for Patient Comorbid Conditions **eAppendix 2.** Ovarian Surgery Coding Definitions
eReferences.

Supplement 2.
Data Sharing Statement
