# Supplementary material for: Factor Analysis of Health Care Access With Ovarian Cancer Surgery and Gynecologic Oncologist Consultation
Source: JAMA Netw Open. 2023 Feb 1;6(2):e2254595. doi: 10.1001/jamanetworkopen.2022.54595 (PMC9892953; doi:10.1001/jamanetworkopen.2022.54595)
Supplement: Supplement 1. — eMethods. eTable 1. Penchansky Model of Health Care Access Dimensions and Example Measures eTable 2. Standardized Factor Loadings and Reliability Test From the 2-Stage 3-Factor Solution for Original and Adjusted Factors eTable 3. Factor Loadings and Reliability Test From the Direct 3 Factor Solution (Single-Stage Approach) eTable 4. Fit Statistics for All Tested Factor Models eTable 5. Invariance Assessment for Model Fit Measures Across Race and Ethnicity Groups for the Final Selected Model eTable 6. Factor Loadings With 95% CIs Using the Maximum Likelihood Method eFigure 1. Participant Flowchart for Hispanic, Non-Hispanic Black, and Non-Hispanic White Patients With Ovarian Cancer, SEER-Medicare Data Set, 2008-2015 eFigure 2. Confirmatory Factor Analysis (CFA) Scree Plots: 2-Stage CFA Approach eAppendix 1. Diagnosis Codes for Patient Comorbid Conditions eAppendix 2. Ovarian Surgery Coding Definitions eReferences. [file jamanetwopen-e2254595-s001.pdf]

Supplemental Online Content

Gupta A, Chen Q, Wilson LE, et al. Factor analysis of health care access with ovarian cancer surgery and gynecologic oncologist consultation. *JAMA Netw Open*. 2023;6(2):e2254595.
doi:10.1001/jamanetworkopen.2022.54595

eMethods.

eTable 1. Penchansky Model of Health Care Access Dimensions and Example Measures

eTable 2. Standardized Factor Loadings and Reliability Test From the 2-Stage 3-Factor Solution for Original and Adjusted Factors

eTable 3. Factor Loadings and Reliability Test From the Direct 3 Factor Solution (Single-Stage Approach)

eTable 4. Fit Statistics for All Tested Factor Models

eTable 5. Invariance Assessment for Model Fit Measures Across Race and Ethnicity Groups for the Final Selected Model

eTable 6. Factor Loadings With 95% CIs Using the Maximum Likelihood Method

eFigure 1. Participant Flowchart for Hispanic, Non-Hispanic Black, and Non-Hispanic White Patients With Ovarian Cancer, SEER-Medicare Data Set, 2008-2015

eFigure 2. Confirmatory Factor Analysis (CFA) Scree Plots: 2-Stage CFA Approach

eAppendix 1. Diagnosis Codes for Patient Comorbid Conditions

eAppendix 2. Ovarian Surgery Coding Definitions

eReferences.

This supplemental material has been provided by the authors to give readers additional information about their work.

© 2023 Gupta A et al. *JAMA Network Open.*

eMethods.

Assignment of primary provider and hospital treatment facility: A patient's primary cancer treatment provider was identified as the provider listed on the highest number of the patient's outpatient, carrier, home health, and hospice claims listing a cancer diagnosis. Physician specialties were determined from Medicare claims files using Health Care Financing Administration (HCFA) specialty codes. Ties between physicians were broken by prioritizing physician specialties of interest (gynecologic oncology, medical oncology, hematology/oncology, or surgical oncology) and claim date closest to the ovarian cancer (OC) diagnosis date. The patient's primary treating hospital in the year the patient was diagnosed was defined as the facility at which the patient had the majority of inpatient and outpatient claims in that calendar year. In the case of ties, priority was given to facilities with records in the SEER-Medicare Hospital File.

Measures of healthcare Affordability: Measures of healthcare Affordability included dual enrollment in Medicaid, census tract-level measures of socioeconomic status (SES), and county-level health insurance coverage. A patient's dual Medicaid enrollment status in the 12 months prior to OC diagnosis was sourced from the SEER-Medicare dataset, as were the following SES indicators of the patient's residential census tract at the time of diagnosis drawn from data from the US Census Bureau's 2010 American Community Survey: median per capita income, percentage of Black residents, percentage of adults 25+ with less than a high school education, percentage of households with incomes below the poverty level, and percentage of adults 25+ with a college degree. Census tract SES characteristics were categorized into quartiles and included as binary variables in models (highest quartile versus lower three quartiles). Federal Information Processing Standards (FIPS) codes for the patient's county and state of residence and the patient's year of diagnosis were used to link to the US Census Bureau's Small Area Health Insurance Estimates 2008-2018 American Community Survey-Based Estimates datasets (https://www.census.gov/data/datasets/time-series/demo/sahie/estimates-acs.html) to obtain the estimated percentage of county residents without health insurance in the year of the patient's diagnosis.

Measures of healthcare Availability: Healthcare Availability metrics for the patient's county and healthcare referral regions were linked to SEER-Medicare data using year of diagnosis, county and state FIPS codes, and patient zip codes from the Area Healthcare Resources File and the Dartmouth Atlas Project. County-level metrics were drawn from the publicly available Area Healthcare Resource Files provided by the Health Resources and Services Administration (https://data.hrsa.gov/data/download). County-level linked measures were calculated as number per 1,000 population and included number of hospitals, number of primary care providers, and number of obstetricians-gynecologists (Ob-Gyns). Hospital referral region (HRR)-level

© 2023 Gupta A et al. *JAMA Network Open.*

availability metrics derived from Medicare and Medicaid data from the Dartmouth Atlas Project (https://atlasdata.dartmouth.edu/downloads) were linked using patient zip code and year of diagnosis. HRR data captures the characteristics of the regional markets for tertiary healthcare systems. HRR-level availability metrics of interest for the patient's year of diagnosis were acute care beds available per 1K population, physicians per 100K population, primary care physicians per 100K population, Hematologists/Oncologists per 100K population, Ob-Gyns per 100K women aged 15-44, percentage of Medicare beneficiaries that died, percentage of beneficiaries seeing a primary care physician (PCP) that year, discharges for ambulatory sensitive conditions per 1K population, hospital discharge 30 day readmission rates, and hospital discharge 30 day return to emergency room (ER) rates. For metrics without data available for each calendar year, the information was imputed from the most proximate year available to the patient's diagnosis within five years. The National Cancer Institute (NCI) hospital file was used to determine facility-associated availability metrics including the hospital's ownership, affiliation with a medical school, NCI Cancer Center designation critical access status, and number of beds in the year of the patient's cancer diagnosis. If the hospital's information was missing in a calendar year, the information was imputed as the highest Availability value for the hospital recorded in the study time period. OC surgical volume for Medicare beneficiaries for each facility was calculated per year by summing the number of Medicare claims among all SEER-Medicare OC patients in the calendar year with a Current Procedural Terminology (CPT) code for an ovarian surgical procedure, allowing 1 surgical claim per patient per day.

Measure selection summary

Area-level statistics were based on the patient's residence at the time of their diagnosis, and when possible, specific to the year of diagnosis. Otherwise, statistics for the closest year available (within 5 years) were utilized (N=7 measures). Ten variables including Census tract-level median household income, percentage of Black residents, percentage of residents aged 25+ with 4 years of college, and patient dual enrollment in Medicaid and Medicare were selected to estimate overall healthcare Affordability. Six variables including patient residence at diagnosis in a metropolitan area, straight line geographic distance in miles from a patient's residence to their main treatment hospital, patient's main treatment hospital's location in an urban or rural area, etc. were abstracted to represent healthcare Accessibility. Nineteen variables chosen to represent healthcare Availability in patient's year of diagnosis include county-level number of gynecologic oncologists per 1K residents, HRR-level 30 day hospital readmission rates, and patient-level main treatment hospital designated as National Cancer Institute (NCI) cancer center.

Parallel analysis for exploratory factor analysis (EFA) and confirmatory factor analysis (CFA)

© 2023 Gupta A et al. *JAMA Network Open.*

Supplemental Table 4 shows the results of fitting all latent variable models. We first compared the EFA models with different sets of factors and the 3-factor CFA models with our final selected model. Then, we calculated the EFA model estimate index for each HCA component and compared the model estimates between the different sets of factors. For model fit measures, to evaluate the goodness-of-fit indices for the models, the comparative fit index (CFI; 0.9 or greater), the Tucker-Lewis Index (TLI; 0.9 or greater), and the standardized root-mean-square residual (SRMR; 0.08 or less) were used.[1,2]

M1 to M4 show the EFA models including all 35 variables with 1-factor to 4-factors structures. The first three models did not fit well based on standard fit statistics (high chi-square/SRMR values and low CFI/TLI). Model 4 with the 4-factor model fit better but showed multiple Heywood cases (negative residuals variance). Model 5 shows the direct CFA with 3 factors. It had the worst fit estimates among all models; this supported the need to modify our model to fit the concept established by Penchansky and Thomas framework of access. Model 6 was our final selected model based on the approach mentioned in our manuscript. It had the best model fit estimates (CFI, TLI, and SRMR).

M7 to M9.2 show the EFA analysis for each component of HCA by selecting only related variables (9 items for Affordability, 6 for Accessibility, and 19 for Availability). Using Mplus, we allowed the factors to be selected ranging from 1 to 3. For Affordability, only 1 factor was recommended (even though our parallel analysis suggested 3 factors). For Accessibility, both 1-factor (M8.1) and 2-factors (M8.2) have great model fits, however only 1 item with a large loading constituted one of the factors for M8.2. Thus, we choose the 1-factor model for Accessibility. Models 9.1 and 9.2 show the results of the Availability EFA 1-factor and 2-factor models. Only up to two factors were recommended. The 2-factor model showed multiple Heywood cases, so we chose the 1-factor model as our selection.

© 2023 Gupta A et al. *JAMA Network Open.*

eTable 1. Penchansky Model of Health Care Access Dimensions and Example Measures

Dimension	Examples of Measure
Affordability<br>Price, willingness and ability to pay for healthcare services	Income
	Insurance type
	Insurance co-pay
	Out-of-pocket cost
	Census tract median household income, poverty
	% Uninsured
Availability<br>Type, quality and volume of healthcare services in relation to patient need	Usual healthcare provider
	Number of hospitals
	Number of gynecologic-oncologists, obstetricians-gynecologists
	Physician specialty
	Hospital ovarian cancer patient volume
	Hospital teaching status
	Hospital quality metrics
Accessibility<br>Location of healthcare services in relation to patients	Travel time (minutes)
	Mode of transport
	Distance to hospital (miles)
	Rural/urban location
Accommodation<br>Organization of healthcare services and resources in relation to patients' ability to accommodate such services	Hospital bed size
	Hospital average wait times
	Access to support services
Acceptability<br>Patient experience, and quality of patient-provider interaction	Trust
	Comfort
	Empathy and respect
	Cultural competence
	Race concordance
	Reputation/credentials
Adapted from Penchansky and Thomas.[3]	

© 2023 Gupta A et al. *JAMA Network Open.*

eTable 2. Standardized Factor Loadings and Reliability Test From the 2-Stage 3-Factor Solution for Original and Adjusted Factors

	Original[a]	Latent factor's composite reliability (Ω) (>0.7)	Latent factor's average variance extracted (AVE) (>0.5)	Adjusted[b]	Latent factor's composite reliability (Ω) (>0.7)	Latent factor's average variance extracted (AVE) (>0.5)
Factor 1						
HRR: Hematologists/oncologists per 100,000 residents (2011)	0.692	0.848	0.636	0.692	0.848	0.637
HRR: Hospital-based physicians per 100,000 residents (2011)	0.577			0.580		
HRR: Primary care physicians per 100,000 residents (2011)	0.806			0.806		
HRR: Total physicians per 100,000 residents (2011)	1			1		
HRR: Surgeons per 100,000 residents (2011)	0.735			0.735		
Factor 2						
Census tract at diagnosis: Percent persons 25+ with at least 4 years of college	0.896	0.876	0.689	0.896	0.876	0.689
Census tract at diagnosis: Median household income	0.897			0.896		
Census tract at diagnosis: Percent persons 25+ with <12 year education	0.714			0.714		
Census tract at diagnosis: Per capita income for Census tract	0.926			0.926		
Census tract at diagnosis: % of households below poverty line	0.683			0.683		
Factor 3						
Patient residence in a metropolitan or metropolitan-adjacent area	0.664	0.394	0.396	0.649	0.798	0.634
Patient lives in metropolitan area	0.861			0.937		
Patient's main hospital is rural primary	0.464			0.401		
County level: # hospitals per 1K residents in year of diagnosis	-0.629					

[a]Original model: root mean square error of approximation (RMSEA) 0.117; comparative fit index (CFI) 0.9; Tucker-Lewis index (TLI) 0.878; standardized root mean squared residual (SRMR) 0.075; Akaike information criterion (AIC) 372369; Bayesian information criterion (BIC) 372688.

[b]Adjusted model: RMSEA 0.117; CFI 0.912; TLI 0.889; SRMR 0.072; AIC 254006; BIC 254304.

© 2023 Gupta A et al. *JAMA Network Open.*

eTable 3. Factor Loadings and Reliability Test From the Direct 3 Factor Solution (Single-Stage Approach)

Factors	Variables	CFA Standardized Factor Loadings	Latent factor’s composite reliability (Ω) (>0.7)	Latent factor’s average variance extracted (AVE) (>0.5)
Factor 1	Patient residence in a metropolitan or metropolitan-adjacent area	0.255	0.777	0.696
	Patient lives in metropolitan area	0.442		
	Patient's main hospital is rural primary	-0.294		
	County level: # gynecologic-oncologists per 1K residents in year of diagnosis	-0.903		
	County level: # hospitals per 1K residents in year of diagnosis	-0.829		
Factor 2	County level: % Uninsured	-0.267	0.357	0.334
	HRR: Hematologists/oncologists per 100,000 residents (2011)	0.749		
	HRR: Hospital-based physicians per 100,000 residents (2011)	0.653		
	HRR: Obstetrician/gynecologists per 100,000 women aged 15-44 (2011)	0.602		
	HRR: Primary care physicians per 100,000 residents (2011)	0.841		
	HRR: Total physicians per 100,000 residents (2011)	1		
	HRR: Surgeons per 100,000 residents (2011)	0.710		
Factor 3	Census tract at diagnosis: % Black residents	-0.353	0.420	0.392
	Census tract at diagnosis: Percent persons 25+ with at least 4 years of college	0.897		
	Census tract at diagnosis: Median household income	0.896		
	Census tract at diagnosis: Percent persons 25+ with <12 year education	0.713		
	Census tract at diagnosis: Per capita income for Census tract	0.921		
	Census tract at diagnosis: % of households below poverty line	0.689		
	HRR: Discharges for ambulatory sensitive conditions per 1K population	-0.308		
	HRR: Percentage of Medicare beneficiaries that died that year	-0.456		
	HRR: 30 day hospital readmission rates	-0.063		
Abbreviation: hospital referral region (HRR)				

© 2023 Gupta A et al. *JAMA Network Open.*

eTable 4. Fit Statistics for All Tested Factor Models

Models	Log-Likelihood	Number of Parameters	BIC	df	χ2	CFI	TLI	SRMR	Note
Factor Analysis									
M1: EFA 1f	-740463	105	1481881	560	180811	0.194	0.144	0.123	
M2: EFA 2f	-706214	139	1413693	526	117312	0.478	0.41	0.114	
M3: EFA 3f	-686650	172	1374865	493	81684	0.637	0.562	0.091	
M4: EFA 4f	-676459	204	1354775	461	63479	0.718	0.636	0.077	*Multiple Heywood cases (negative residuals)
M5: CFA 3f	-744612	102	1490153	563	184390	0.178	0.132	0.358	
**M6: Modified CFA 3f**	**-165446**	**45**	**331302**	**74**	**9150**	**0.9**	**0.878**	**0.075**	
EFA for each component									
**M7: EFA-Affordability 1f**	**-235426**	**30**	**471126**	**35**	**11499**	**0.677**	**0.585**	**0.082**	*Mplus only suggested 1 factor
**M8.1: EFA-Accessibility 1f**	**-67591**	**18**	**135346**	**9**	**204**	**0.94**	**0.9**	**0.037**	
M8.2: EFA-Accessibility 2f	-67245	23	134700	4	10	0.998	0.993	0.006	*Only one variable loaded on factor 2
**M9.1: EFA-Availability 1f**	**-480776**	**57**	**962072**	**152**	**103993**	**0.281**	**0.191**	**0.169**	
M9.2: EFA-Availability 2f	-471297	75	943277	134	87634	0.394	0.227	0.146	*Multiple Heywood cases
Abbreviations: Bayesian information criterion (BIC); degrees of freedom (df); comparative fit index (CFI); Tucker-Lewis index (TLI); standardized root mean squared residual (SRMR)									

© 2023 Gupta A et al. *JAMA Network Open.*

eTable 5. Invariance Assessment for Model Fit Measures Across Race and Ethnicity Groups for the Final Selected Model

Models	No. Observations	CFI	TLI	SRMR	RMSEA
All	8987	0.9	0.878	0.075	0.117
Hispanic	553	0.901	0.879	0.073	0.123
NH-Black	612	0.895	0.871	0.081	0.12
NH-White	7822	0.899	0.876	0.074	0.117
Abbreviations: comparative fit index (CFI); Tucker-Lewis index (TLI); standardized root mean squared residual (SRMR); non-Hispanic (NH)					

© 2023 Gupta A et al. *JAMA Network Open.*

eTable 6. Factor Loadings With 95% CIs Using the Maximum Likelihood Method

Factors	Variables	Standardized Factor Loadings (95% CI)		
Factor 1 (Availability)	HRR: Hematologists/oncologists per 100,000 residents (2011)			0.692 (0.687, 0.697)
	HRR: Hospital-based physicians per 100,000 residents (2011)			0.577 (0.574, 0.580)
	HRR: Primary care physicians per 100,000 residents (2011)			0.806 (0.803, 0.809)
	HRR: Total physicians per 100,000 residents (2011)			1.000 (0.998, 1.002)
	HRR: Surgeons per 100,000 residents (2011)			0.735 (0.731, 0.739)
Factor 2 (Affordability)	Census tract at diagnosis: Percent residents 25+ with at least 4 years of college	0.896 (0.893, 0.899)		
	Census tract at diagnosis: Median household income	0.897 (0.894, 0.900)		
	Census tract at diagnosis: Percent residents 25+ with <12 years education	-0.714 (-0.708, -0.720)		
	Census tract at diagnosis: Mean per capita income	0.926 (0.924, 0.928)		
	Census tract at diagnosis: Percentage households below poverty line	-0.683 (-0.677, -0.689)		
Factor 3 (Accessibility)	Patient residence in a metropolitan or metropolitan-adjacent area		0.664 (0.657, 0.671)	
	Patient lives in metropolitan area		0.861 (0.855, 0.867)	
	Patient's main hospital is designated rural primary hospital		-0.464 (-0.454, -0.474)	
	County level: # hospitals per 1K residents in year of diagnosis		-0.629 (-0.621, -0.637)	
Abbreviations: hospital referral region (HRR); 95% Confidence Interval (95% CI)				

© 2023 Gupta A et al. *JAMA Network Open.*

eFigure 1. Participant Flowchart for Hispanic, Non-Hispanic Black, and Non-Hispanic White Patients With Ovarian Cancer, SEER-Medicare Data Set, 2008-2015

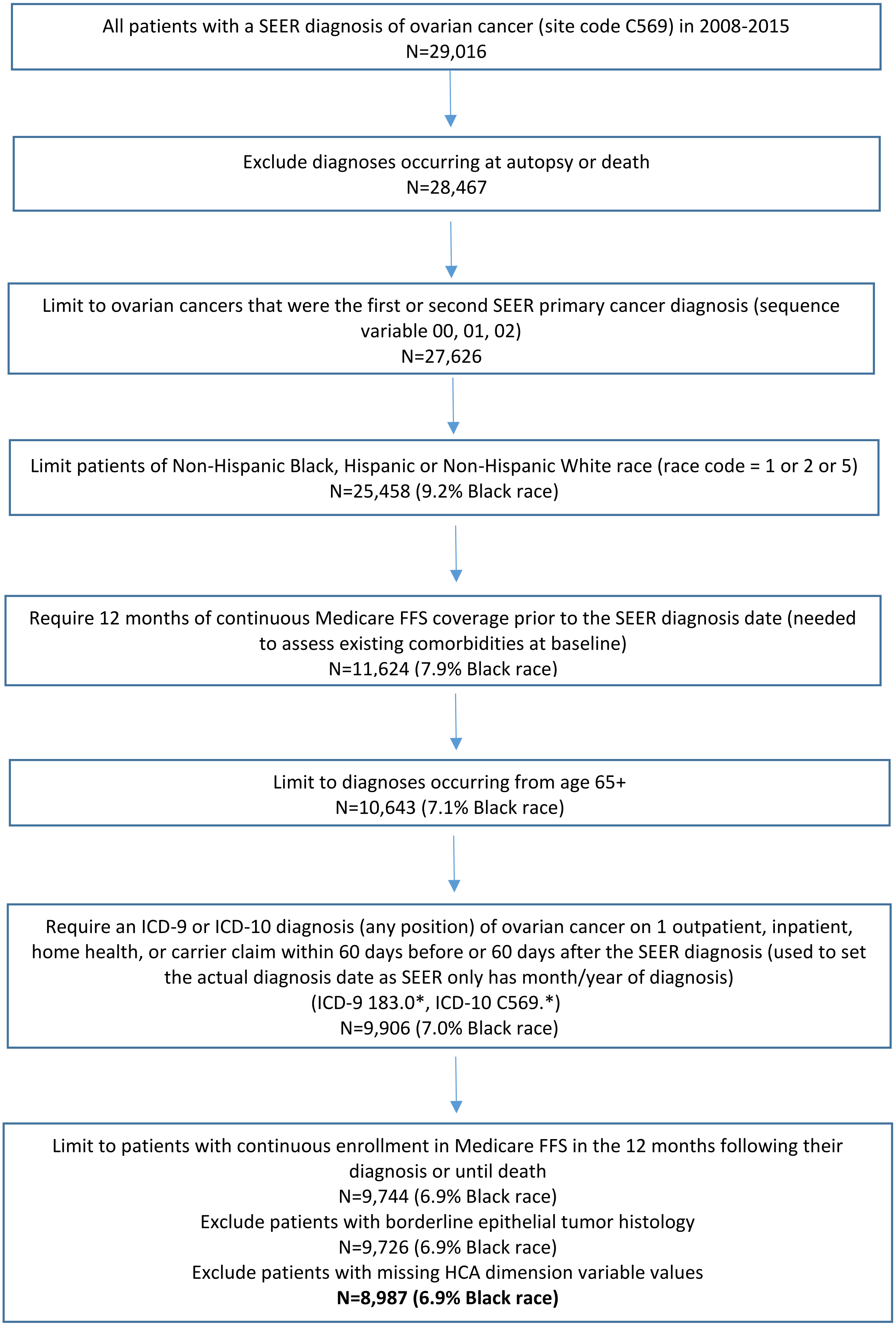

© 2023 Gupta A et al. *JAMA Network Open.*

eFigure 2. Confirmatory Factor Analysis (CFA) Scree Plots: 2-Stage CFA Approach

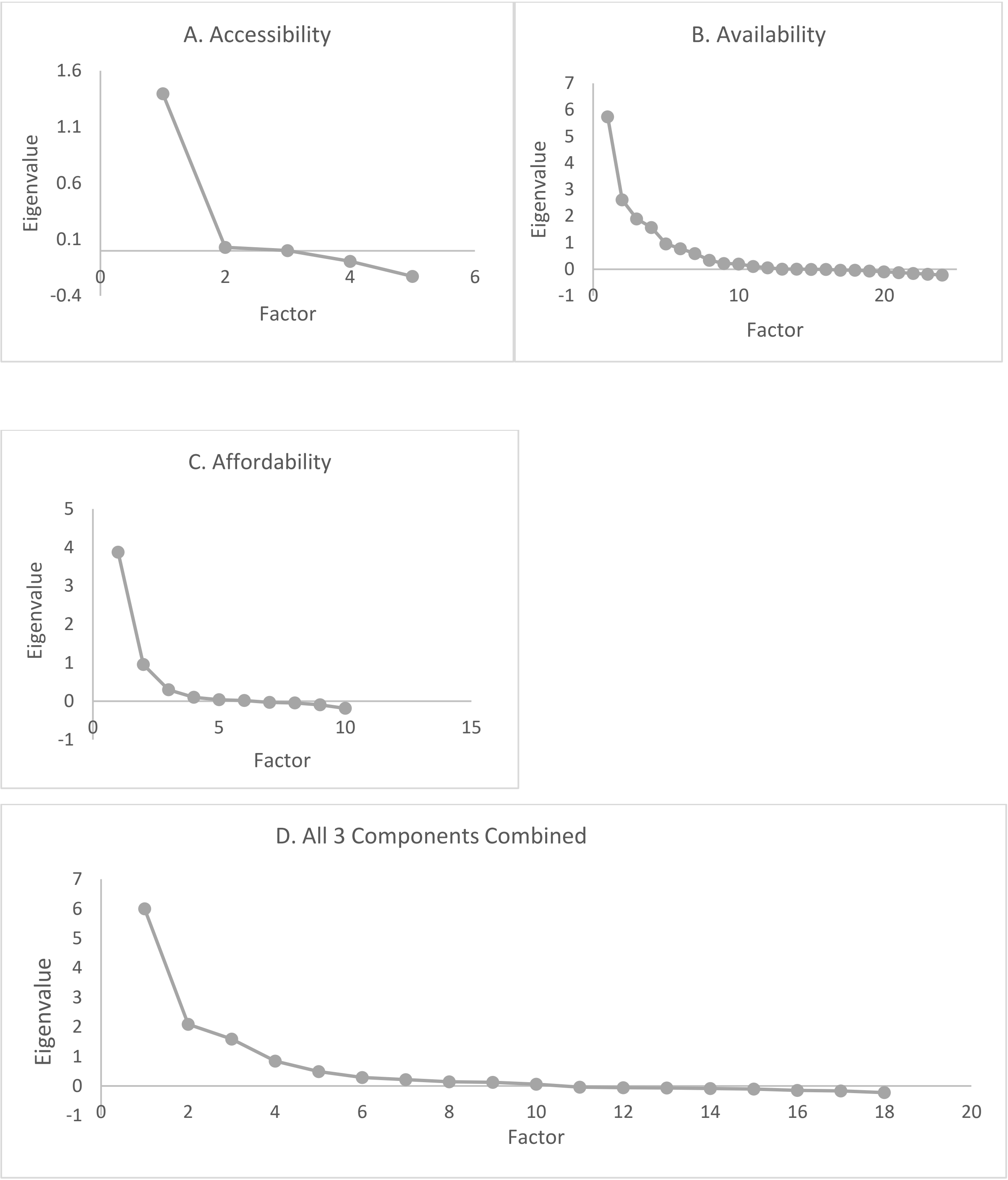

© 2023 Gupta A et al. *JAMA Network Open.*

eAppendix 1. Diagnosis Codes for Patient Comorbid Conditions

Baseline Deyo-Charlson Comorbidity Index	The Deyo-Charlson Comorbidity will be created during the baseline period based on the following score:<br>o 1 each: Myocardial infarction, congestive heart failure, peripheral vascular disease, dementia, cerebrovascular disease, chronic pulmonary disease, rheumatologic disease, peptic ulcer disease, mild liver disease, diabetes (mild to moderate).<br>o 2 each: Hemiplegia or paraplegia, renal disease, diabetes with complication, any malignancy (leukemia, lymphoma).<br>o 3 each: Moderate or severe liver disease.<br><br>6 each: Malignant tumor, acquired immunodeficiency syndrome (AIDS).
Myocardial infarction	ICD-9-CM codes: 410.*, 412.*<br>ICD-10-CM codes: I21.*, I22.*, I25.2*
Congestive heart failure	ICD-9-CM codes: 398.91, 402.01, 402.11, 402.91, 404.01, 404.03, 404.11, 404.13, 404.91, 404.93, 425.4*, 425.5*, 425.7*, 425.8*, 425.9*, 428.*<br>ICD-10-CM codes: I09.81, I11.0*, I13.0*, I13.2*, I42.0*, I42.5*, I42.6*, I42.7*, I42.8*, I42.9*, I43.*, I50.*
Peripheral vascular disease	ICD-9-CM codes:<br>093.0*, 437.3*, 440.*, 441.*, 443.1*, 443.2*, 443.8*, 443.9*, 447.1*, 557.1*, 557.9*, V43.4*<br>ICD-10-CM codes:<br>A52.01, E08.51, E08.52, E09.51, E09.52, E10.51, E10.52, E11.51, E11.52, E13.51, E13.52, I67.0*, I67.1*, I70.*, I71.*, I73.1*, I73.8*, I73.9*, I77.7*, I79.*, K55.1*, K55.8*, K55.9*, Z95.82
Hypertension	ICD-9-CM codes:<br>401.*, 402.*, 403.*, 404.*, 405.*, 437.2*<br>ICD-10-CM codes:<br>I10.*, I11.*, I12.*, I13.*, I15.0*, I15.2*, I15.8*, I15.9*, I16.*, I67.4*
Dementia	ICD-9-CM codes:<br>290.*, 294.1*, 331.2*<br>ICD-10-CM codes:<br>F01.*, F02.*, F03.9*, G31.1*
Cerebrovascular disease	ICD-9-CM codes:<br>362.34, 430.*, 431.*, 432.*, 433.*, 434.*, 435.*, 436.*, 437.*, 438.*<br>ICD-10-CM codes:<br>G45.0*, G45.1*, G45.2*, G45.4*, G45.8*, G45.9*, G46.*, H34.0*, I60.*, I61.*, I62.*, I63.*, I65.*, I66.*, I67.1*, I67.2*, I67.4*, I67.5*, I67.6*, I67.7*, I67.81, I67.82, I67.84, I67.89, I67.9*, I68.*, I69.*
Chronic pulmonary disease	ICD-9-CM codes:<br>416.8*, 416.9*, 490.*, 491.*, 492.*, 493.*, 494.*, 495.*, 496.*, 500.*, 501.*, 502.*, 503.*, 504.*, 505.*, 506.4*, 508.1*, 508.8*<br>ICD-10-CM codes:<br>I27.2*, I27.81, I27.89, I27.9*, J40.*, J41.*, J42.*, J43.*, J44.*, J45.2*, J45.3*, J45.4*, J45.5*', 'J45.90, J45.99, J47.*, J60.*, J61.*, J62.*, J63.*, J64.*, J65.*, J66.*, J67.*, J68.4*, J70.1*, J70.2*, J70.3*, J70.4*, J70.8*
Rheumatologic disease	ICD-9-CM codes:<br>446.5*, 710.0*, 710.1*, 710.2*, 710.3*, 710.4*, 714.0*, 714.1*, 714.2*, 714.8*, 725.*<br>ICD-10-CM codes:<br>M05.*, M06.*, M31.5*, M31.6*, M32.*, M33.*, M34.*, M35.0*, M35.3*, M36.0*

© 2023 Gupta A et al. *JAMA Network Open.*

Peptic ulcer disease	ICD-9-CM codes:<br>531.*, 532.*, 533.*, 534.*<br>ICD-10-CM codes:<br>K25.*, K26.*, K27.*, K28.*
Mild liver disease	ICD-9-CM codes:<br>070.22, 070.23, 070.32, 070.33, 070.44, 070.54, 070.6*, 070.9*, 570.*, 571.*, 573.3*, 573.4*, 573.8*, 573.9*, V42.7*<br>ICD-10-CM codes:<br>B17.9*, B18.0*, B18.1*, B18.2*, B19.0*, B19.9*, K70.0*, K70.1*, K70.2*, K70.3*, K70.40, K70.9*, K71.0*, K71.10, K71.2*, K71.3*, K71.4*, K71.5*, K71.6*, K71.7*, K71.8*, K71.9*, K72.00, K73.*, K74.0*, K74.1*, K74.2*, K74.3*, K74.4*, K74.5*, K74.6*, K75.2*, K75.3*, K75.4*, K75.8*, K75.9*, K76.0*, K76.1*, K76.2*, K76.3*, K76.4*, K76.5*, K76.89, K76.9*, K77.*, Z48.23, Z94.4*
Diabetes (mild to moderate)	ICD-9-CM codes:<br>250.0*, 250.1*, 250.2*, 250.3*, 250.8*, 250.9*<br>ICD-10-CM codes:<br>E10.1*, E10.618, E10.62, E10.63, E10.64, E10.65, E10.69, E10.8*, E10.9*, E11.0*, E11.1*, E11.618, E11.62, E11.63, E11.64, E11.65, E11.69, E11.8*, E119, E13.00, E13.01, E13.10, E13.11, E13.618, E13.62, E13.63, E13.64, E13.65, E13.69, E13.8*, E13.9*
Hemiplegia or paraplegia	ICD-9-CM codes:<br>334.1*, 342.*, 343.*, 344.0*, 344.1*, 344.2*, 344.3*, 344.4*, 344.5*, 344.6*, 344.9*<br>ICD-10-CM codes:<br>G04.1*, G11.4*, G80.*, G81.*, G82.*, G83.0*, G83.1*, G83.2*, G83.3*, G83.4*, G83.9*
End Stage Renal Disease	585.6, N18.6 or ESRD eligibility flag
Diabetes with complication	ICD-9-CM codes:<br>250.4*, 250.5*, 250.6*, 250.7*<br>ICD-10-CM codes:<br>E10.2*, E10.3*, E10.4*, E10.5*, E10.610, E11.2*, E11.3*, E11.4*, E11.5*, E11.610, E13.2*, E13.3*, E13.4*, E13.5*, E13.610
Moderate or severe liver disease	ICD-9-CM codes:<br>456.0*, 456.1*, 456.2*, 572.2*, 572.3*, 572.4*, 572.8*<br>ICD-10-CM codes:<br>I85.*, K70.41, K71.11, K72.01, K72.1*, K72.9*, K76.6*, K76.7*
Acquired immunodeficienc y syndrome (AIDS)	ICD-9-CM codes:<br>042.*, 043.*, 044.*<br>ICD-10-CM codes:<br>B20.*

© 2023 Gupta A et al. *JAMA Network Open.*

eAppendix 2. Ovarian Surgery Coding Definitions

Billing Codes for Surgery	
**International Classification of Disease, ninth revision (ICD-9 and ICD-10 procedure codes)**	
ICD-9: 54.4	Omentectomy, excision, destruction peritoneal tissue
ICD-10-Mapping from CMS GEMS:<br>0D5U0ZZ, 0D5U3ZZ, 0D5U4ZZ, 0D5V0ZZ, 0D5V3ZZ, 0D5V4ZZ, 0D5W0ZZ, 0D5W3ZZ, 0D5W4ZZ, 0DBU0ZZ, 0DBU3ZZ, 0DBU4ZZ<br>0DBV0ZZ 0DBV3ZZ, 0DBV4ZZ, 0DBW0ZZ, 0DBW3ZZ, 0DBW4ZZ, 0DTU0ZZ, 0DTU4ZZ, 0WBH0ZZ, 0WBH3ZZ, 0WBH4ZZ	
ICD-9: 65.2x	Wedge resection or partial excision of ovary
ICD-10, GEMS:<br>0U900ZZ, 0U903ZZ, 0U910ZZ, 0U913ZZ, 0U920ZZ, 0U923ZZ, 0UB00ZZ, 0UB03ZZ, 0UB07ZZ, 0UB08ZZ, 0UB10ZZ, 0UB13ZZ, 0UB17ZZ, 0UB18ZZ, 0UB20ZZ, 0UB23ZZ, 0UB27ZZ, 0UB28ZZ, 0U904ZZ, 0U914ZZ, 0U924ZZ, 0UB04ZZ, 0UB14ZZ, 0UB24ZZ, 0U504ZZ, 0U514ZZ, 0U524ZZ, 0UB04ZZ, 0UB14ZZ, 0UB24ZZ, 0U500ZZ, 0U503ZZ, 0U508ZZ, 0U510ZZ, 0U513ZZ, 0U518ZZ, 0U520ZZ, 0U523ZZ, 0U528ZZ, 0U800ZZ, 0U803ZZ, 0U810ZZ, 0U813ZZ, 0U820ZZ, 0U823ZZ, 0UB00ZZ, 0UB03ZZ, 0UB10ZZ, 0UB13ZZ, 0UB20ZZ, 0UB23ZZ	
ICD-9: 65.3x	Unilateral oophorectomy
ICD-10, GEMS:<br>0UT04ZZ, 0UT14ZZ, 0UT00ZZ, 0UT07ZZ, 0UT08ZZ, 0UT0FZZ, 0UT10ZZ, 0UT17ZZ, 0UT18ZZ, 0UT1FZZ	
ICD-9: 65.4x	Bilateral oophorectomy
ICD-10, GEMS:<br>0UT04ZZ, 0UT14ZZ, 0UT54ZZ, 0UT64ZZ, 0UT00ZZ, 0UT10ZZ, 0UT50ZZ, 0UT60ZZ	
ICD-9: 65.51-65.54	Other removal of ovaries
ICD-10, GEMS:<br>0UT20ZZ, 0UT27ZZ, 0UT28ZZ, 0UT2FZZ, 0UT00ZZ, 0UT07ZZ, 0UT08ZZ, 0UT0FZZ, 0UT10ZZ, 0UT17ZZ, 0UT18ZZ, 0UT1FZZ, 0UT24ZZ, 0UT04ZZ, 0UT14ZZ	
ICD-9: 65.6x	Bilateral salpingoopherectomy
ICD-10, GEMS:<br>0UT20ZZ, 0UT70ZZ, 0UT00ZZ, 0UT10ZZ, 0UT50ZZ, 0UT60ZZ, 0UT24ZZ, 0UT74ZZ, 0UT04ZZ, 0UT14ZZ, 0UT54ZZ, 0UT64ZZ	
ICD-9: 66.63, 66.69	Bilateral/other partial salpingectomy
ICD-10, GEMS:<br>0UB70ZZ, 0UB73ZZ, 0UB74ZZ, 0UB77ZZ, 0UB78ZZ, 0UB50ZZ, 0UB53ZZ, 0UB54ZZ, 0UB57ZZ, 0UB58ZZ, 0UB60ZZ, 0UB63ZZ,<br>0UB64ZZ, 0UB67ZZ, 0UB68ZZ	
ICD-9: 68.8	Pelvic exenteration
ICD-10, GEMS:	

© 2023 Gupta A et al. *JAMA Network Open.*

**Billing Codes for Surgery**		
0UB70ZZ, 0UB73ZZ, 0UB74ZZ, 0UB77ZZ, 0UB78ZZ,<br>0UB50ZZ, 0UB53ZZ, 0UB54ZZ, 0UB57ZZ, 0UB58ZZ,<br>0UB60ZZ, 0UB63ZZ, 0UB64ZZ, 0UB67ZZ, 0UB68ZZ		
ICD-9: 68.3-68.7, 68.9, 68.59		Hysterectomy
ICD-10, GEMS:<br>0UT94ZL, 0UT90ZL, 0UT94ZZ, 0UTC4ZZ, 0UT90ZZ,<br>0UTC0ZZ, 0UT9FZL, 0UT9FZZ, 0UTC4ZZ, 0UT97ZL,<br>0UT97ZZ, 0UT98ZL, 0UT98ZZ, 0UTC7ZZ, 0UTC8ZZ,<br>0UT44ZZ, 0UT94ZZ, 0UTC4ZZ, 0UT40ZZ, 0UT90ZZ,<br>0UTC0ZZ, 0UT44ZZ, 0UT9FZZ, 0UTC4ZZ, 0UT47ZZ,<br>0UT48ZZ, 0UT97ZZ, 0UT98ZZ, 0UTC7ZZ, 0UTC8ZZ		
ICD-9: 70.32		Excision/destruction cul de sac lesion
ICD-10, GEMS:<br>0U5F0ZZ, 0U5F3ZZ, 0U5F4ZZ, 0U5F7ZZ, 0U5F8ZZ,<br>0UBF0ZZ, 0UBF3ZZ, 0UBF4ZZ, 0UBF7ZZ, 0UBF8ZZ		
**Common Procedural Terminology (CPT) Codes**		
56303		Laparoscopy with excision of ovary or peritoneum
56307		Laparoscopic oophorectomy +/- salpingectomy
56308		Laparoscopy and vaginal hysterectomy +/- salpingo-oophorectomy
57531		Para-aortic lymph node sampling +/- salpingo-oophorectomy
58150		TAH +/- salpingo-oophorectomy
58152		TAH with colpo-urethrocystopexy +/- salpingo-oophorectomy
58180		Subtotal hysterectomy +/- salpingo-oophorectomy
58200		TAH with para-aortic and pelvic lymph node sampling +/- salpingo-oophorectomy
58210		Radical Hysterectomy
58240		Pelvic exenteration, including colostomy
58262		Vaginal Hysterectomy +/- salpingo-oophorectomy
58263		Vaginal Hysterectomy with repair of enterocele +/- salpingo-oophorectomy
58720		Salpingo-oophorectomy, complete or partial, unilateral or bilateral
58920		Wedge resection of ovary
58940		Oophorectomy, partial or total, unilateral or bilateral
58943		Oophorectomy, partial or total, unilateral or bilateral; for ovarian malignancy, with para-aortic and pelvic lymph node biopsies, peritoneal washings, peritoneal biopsies, diaphragmatic assessment, with or without salpingectomy(s), with or without omentectomy
58950		Resection of ovarian malignancy with bilateral salpingo-oophorectomy and omentectomy
58951		Resection of ovarian malignancy with bilateral salpingo-oophorectomy and omentectomy, with abdominal hysterectomy, pelvic and limited para-aortic lymphadenectomy)

© 2023 Gupta A et al. *JAMA Network Open.*

Billing Codes for Surgery		
58952		Resection of ovarian malignancy with bilateral salpingo-oophorectomy and omentectomy, with radical dissection for debulking
58953		Bilateral salpingo-oophorectomy with omentectomy, total abdominal hysterectomy and radical dissection for debulking
58954		Bilateral salpingo-oophorectomy with omentectomy, total abdominal hysterectomy and radical dissection for debulking, with pelvic lymphadenectomy and limited para-aortic lymphadenectomy)
58960		Laparotomy for staging or restaging of ovarian, tubal or primary peritoneal malignancy (second look) with or without omentectomy, peritoneal washing, biopsy of abdominal and pelvic peritoneum, diphragamatic assessment with pelvic and limited per-aortic lymphadenectomy

© 2023 Gupta A et al. *JAMA Network Open.*

eReferences.

1. Bentler PM. Comparative fit indexes in structural models. *Psychological bulletin.* 1990;107(2):238-246.
2. Quintana SM, Maxwell SE. Implications of Recent Developments in Structural Equation Modeling for Counseling Psychology. *The Counseling Psychologist.* 1999;27(4):485-527.
3. Penchansky R, Thomas JW. The concept of access: definition and relationship to consumer satisfaction. *Medical care.* 1981;19(2):127-140.

© 2023 Gupta A et al. *JAMA Network Open.*
