# Supplementary material for: Factor Analysis of Health Care Access With Ovarian Cancer Surgery and Gynecologic Oncologist Consultation
Source: JAMA Netw Open. 2023 Feb 1;6(2):e2254595. doi: 10.1001/jamanetworkopen.2022.54595 (PMC9892953; doi:10.1001/jamanetworkopen.2022.54595)
Supplement: Supplement 2. — Data Sharing Statement [file jamanetwopen-e2254595-s002.pdf]

Data Sharing Statement

Gupta. Factor Analysis of Health Care Access With Ovarian Cancer Surgery and Gynecologic Oncologist Consultation. *JAMA Netw Open*. Published February 01, 2023. doi:10.1001/jamanetworkopen.2022.54595

Data
Data available: No

Additional Information
Explanation for why data not available: The SEER-Medicare database is owned and managed by the National Cancer Institute. Information on how to obtain these data is available here: https://healthcaredelivery.cancer.gov/seermedicare/obtain/.
